# The Effects of Lipid Membranes, Crowding and Osmolytes on the Aggregation, and Fibrillation Propensity of Human IAPP

**DOI:** 10.1155/2015/849017

**Published:** 2015-10-25

**Authors:** Mimi Gao, Roland Winter

**Affiliations:** Physical Chemistry I-Biophysical Chemistry, Department of Chemistry and Chemical Biology, TU Dortmund, Otto-Hahn Street 6, 44227 Dortmund, Germany

## Abstract

Type 2 diabetes mellitus (T2DM) is an age-related and metabolic disease. Its development is hallmarked, among others, by the dysfunction and degeneration of *β*-cells of the pancreatic islets of Langerhans. The major pathological characteristic thereby is the formation of extracellular amyloid deposits consisting of the islet amyloid polypeptide (IAPP). The process of human IAPP (hIAPP) self-association, and the intermediate structures formed as well as the interaction of hIAPP with membrane systems seem to be, at least to a major extent, responsible for the cytotoxicity. Here we present a summary and comparison of the amyloidogenic propensities of hIAPP in bulk solution and in the presence of various neutral and charged lipid bilayer systems as well as biological membranes. We also discuss the cellular effects of macromolecular crowding and osmolytes on the aggregation pathway of hIAPP. Understanding the influence of different cellular factors on hIAPP aggregation will provide more insight into the onset of T2DM and help to develop novel therapeutic strategies.

## 1. Introduction

Type 2 diabetes mellitus (T2DM) is a metabolic disease that affects over 340 million people worldwide. It is defined by the two hallmarks, insulin resistance and pancreatic *β*-cell failure. The dysfunction and degeneration of pancreatic *β*-cells are caused amongst others by the formation and deposition of extracellular amyloid plaques [1–4]. Such amyloid deposits were described already in 1901 [5, 6]. However, its main amyloidogenic component human islet amyloid polypeptide (hIAPP), also named amylin, was extracted and sequenced 85 years later [7, 8]. Therefore, T2DM belongs to the protein misfolding diseases, also known as proteopathies, which are associated with abnormal accumulation of insoluble fibrillar protein aggregates in tissues and organs. Although distinct proteins are involved in the formation of those deposits in different diseases such as Alzheimer's disease, Parkinson's disease, Huntington's disease and T2DM, amyloids feature a common morphology with cross-*β*-sheets as secondary structure [9–12]. Some peptides, including peptide hormones, show a tendency to aggregation due to their small size, lack of secondary structure as well as their appearance at high local concentrations [13, 14]. During aggregation, specific species such as monomers, oligomers and fibrils can be observed at different stages. In the past it was believed that the fibrillar deposits are the toxic species and are responsible for the pathological phenotype of the disease because they are found in* post mortem* organs or tissues.

Nowadays, there is much evidence that the aggregation process itself or even the intermediate species are cytotoxic, whereas the final fibrillar aggregates and inclusions, respectively, may even have protective functions [11, 15–17]. However, fibrils are the best studied species due to their low solubility and high stability. The* in vitro* formed fibrils consist of several proto-fibrils which are twisted around each other and feature intermolecular *β*-sheets perpendicular to the fibril axis. This characteristic conformation is also known as cross-*β*-sheet structure [9–12].

**Figure 1:**
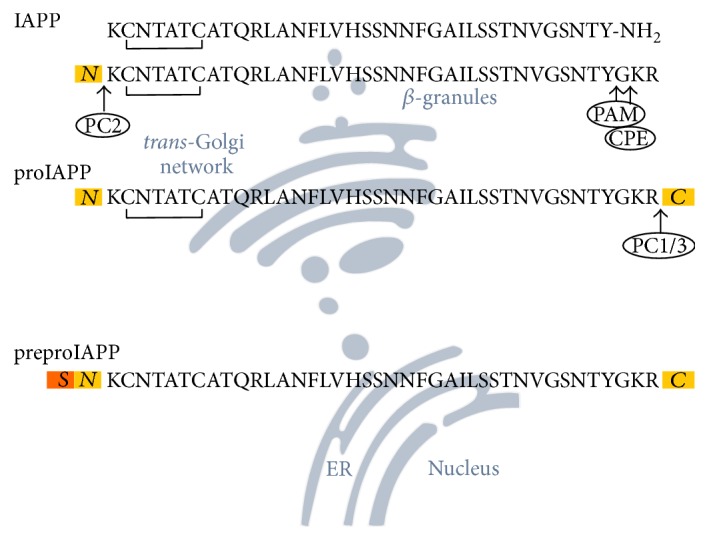
Posttranslational modification of hIAPP through the secretory pathway in islet *β*-cells. Upon expression of the preproIAPP, a disulfide bond is formed at the ER and the N-terminal signal peptide (*S*: MGILKLQVFLIVLSVALNHLKA) is cleaved after transport to the* trans*-Golgi network. The cleavage of the C-terminus fragment (*C*: NAVEVLKREPLNYLPL) from proIAPP is catalyzed by PC1/3. PC2 removes the N-terminal fragment (*N*: TPIESHQVEKR) of proIAPP within the secretory vesicles. The remaining basic residues at the C-terminus are removed by the action of CPE. The final step of removal of Gly38 and amidation of IAPP at the C-terminus is realized by the PAM complex.

IAPP is a 37 amino-acid residues long peptide hormone, which is coproduced and cosecreted along with insulin through the secretory pathway in *β*-cell in a ratio of 1 : 100, but can increase to 1 : 20 in case of T2DM [18]. During protein translation, hIAPP is processed and modified (Figure 1). The hIAPP gene is expressed as an 89 amino-acid residue long preproIAPP. The 20 amino-acid residue long signal peptide, which is located at the N-terminus, guides the protein from the endoplasmic reticulum (ER) to the* trans*-Golgi network. During that transport a disulfide bridge between two cysteine residues is formed. Arrived at the* trans*-Golgi network, the signal peptide is cleaved resulting in a 67 amino-acid residue long proIAPP, which is further processed by prohormone convertase (PC) enzymes. First, the 16 C-terminal amino acids are cleaved by PC1/3 in the* trans*-Golgi network. Next, in the secretory vesicles, PC2 cleaves the 11 N-terminal amino acids. Finally, carboxypeptidase E (CPE) catalyzes the cleavage of the two C-terminal basic amino acids and activates the peptidyl amidating monooxygenase (PAM) complex which operates the cleavage of glycine at position 38 and the amidation of tyrosine at position 37 [1, 19–22].

Contradictory to the concept of folding funnels, the monomeric hIAPP is intrinsically disordered and thus features numerous flexible and random coil conformations [23–25] with a transient amphipathic helix in the N-terminal region [26, 27]. The IAPP22–27 region has been shown to be essential for amyloid formation [28]. It has been suggested that aggregated IAPP is folded into a double *β*-hairpin with three *β*-strands between residues 12 and 37 [29]. However, an alternative atomistic structural model of a single *β*-hairpin has been obtained from three independent studies based on nuclear magnetic resonance (NMR), electron paramagnetic resonance (EPR) and X-ray diffraction approaches [30–32], but they differ in the details of the monomeric folding and the packing of the peptide within the fibrils. Principally, the polymorphic nature of amyloid fibrils provides the possibility that different monomeric conformations and various sets of interresidue interactions within the fibrils can coexist [33]. Interestingly, amyloid formation of IAPP does not occur in every mammalian species, although its primary structure is well conserved through evolution. For example, pathological deposition of IAPP amyloids cannot be found in the islets of Langerhans of rodents [1]. The main reason for this is proline mutations (acting as *β*-sheet breaker) in the most fibrillogenic IAPP20–29 region.

Similar to its monomeric structure, the effect and exact physiological functions of hIAPP are contradictory and still under debate [1]. Due to its cosecretion with insulin, it may act as a hormone regulating the glucose homeostasis. Despite the difficulty to distinguish between its physiological and pathophysiological effects, two fundamental physiological roles of hIAPP have been determined. First, it acts as an auto- or paracrine molecule in the islets of Langerhans regulating the secretion of insulin and glucagon. However, the results are ambiguous, ranging from stimulation via no effect through to inhibition [34–42]. This phenomenon might be explained by the fact that monomeric hIAPP as an intrinsically disordered protein (IDP) features conformational diversity and thus interacts with its target differently depending on its conformation. Second, hIAPP functions as a hormone for the central nervous system. As an anorectic, it reduces the caloric intake and the meal duration [43]. Moreover, inhibitory effects of hIAPP on gastric emptying and bone resorption have been reported [44].

The clear link between islet amyloid deposition and reduction of the *β*-cell mass gives rise to the pathophysiological effects of hIAPP. An increased expression level and aggregation of hIAPP have been reported to cause dysfunctions and death of *β*-cells at different subcellular levels. An inductive effect of hIAPP on the unfolding protein response (UPR) within the ER compartment has been detected when the expression level of hIAPP is upregulated. However, contrary findings have also been reported [45, 46]. Therefore, the ER stress and its role in IAPP toxicity are controversial and are still an open question. Additionally, accumulation of polyubiquitinated proteins and autophagosomes have been found in *β*-cells from T2DM patients at autopsy, indicating dysfunction of the two major intracellular protein degradation systems ubiquitin-proteasome system (UPS) [47] and autophagy [48–51]. There is also evidence that hIAPP contributes to islet inflammation by being internalized in macrophages in its aggregated state in order to activate the Nlrp3 inflammasome and thus the production of the pathogenic cytokine IL-1*β* [52–54].

**Figure 2:**
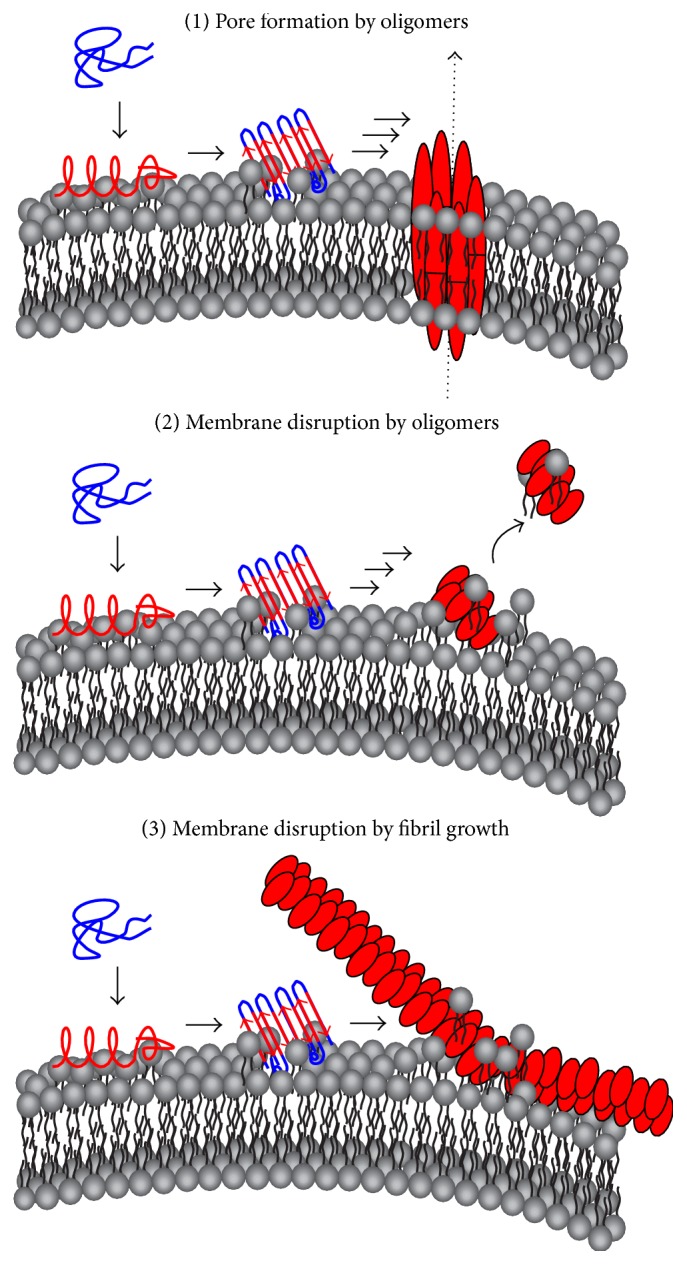
Three different, but not exclusive proposed mechanisms of membrane disruption by hIAPP and its aggregation. Upon binding of hIAPP with the membrane, formation of *α*-helical structure within monomeric hIAPP is favored due to its amphipathic nature. Initial irritation of the membrane integrity occurs. Self-assembly of hIAPP results in the formation of *β*-sheets and causes deeper membrane disruption. The further formation of oligomers leads to enhanced membrane disruption and disintegration either by forming pores within the lipid bilayers (1) or simply by extracting lipids from the membrane (2). At a later stage, the growth of fibrils (3) has also been observed to be responsible for membrane disruption.

An additional major pathophysiological effect of hIAPP is its interaction with membranes.* In vitro* studies show that hIAPP fibrillation is membrane-mediated, especially in the presence of anionic lipids [23, 55–59]. Exogenous exposure of pancreatic *β*-cells (INS-1) to hIAPP via culture medium causes dysfunction of mitochondria and finally death of cells [60, 61]. There is much evidence that hIAPP can escape from the secretory pathway by attacking the vesicle membrane [62–67]. Thus, the hIAPP-mediated cytotoxicity is proposed to be initiated by intracellular oligomerization and fibrillation and presumably caused by disrupting membrane integrity of different cellular compartments, but its mechanism is still under debate. Three general, but not exclusive theories of membrane disruption by hIAPP have been developed (Figure 2). They might act in tandem. (1) The nonspecific model, where the membrane integrity is disturbed by fibril growth on the membrane. In a detergent-like mechanism hIAPP fibrillation causes large-scale defects in the lipid bilayer, resulting in membrane thinning and fragmentation, accompanied by increased membrane conductance [68–73]. (2) Binding of monomeric hIAPP to the membrane facilitates the structural transition of hIAPP from a disordered structure into a partially *α*-helical conformation, followed by oligomerization. The hydrophobic and membrane permeable* on-pathway* oligomers represent the toxic species in this case [17, 26, 74]. (3) Electrophysiological measurements and small molecule selectivity support the pore theory, where hIAPP forms ion channel-like pores (“barrel stave”) within the membrane, leading to deficient ion homeostasis [75–79].

However, the relationship between the formation of islet amyloid and the onset of T2DM is still largely unknown [1]. The question if hIAPP aggregation is a cause for *β*-cell dysfunction and destruction or just a consequence remains unanswered. In secretory vesicles, hIAPP exists at high concentrations (mM range).* In vitro* at those concentrations, hIAPP would rapidly aggregate. This leads to the suggestion that hIAPP has to be stabilized to prevent rapid aggregation* in vivo*. It has been shown that insulin, but not proinsulin, is able to inhibit hIAPP fibril formation* in vitro* by forming heteromolecular complexes. Therefore, deficient insulin processing would prevent this protective interaction and lead to hIAPP aggregation [80–82]. Moreover, age-related changes of environmental factors (pH, salt concentration, chemical modifications, and changes in lipid composition) and protein homeostasis could also lead to destabilization of monomeric hIAPP [80, 83, 84]. However, the exact factors that are responsible for hIAPP aggregation are still poorly understood.

*In vitro*, hIAPP fibrillogenesis has been thoroughly studied by varying salt [85, 86], pH [84] and temperature [87]. On the other hand, hIAPP transgenic mice have been developed to study the consequence of hIAPP aggregation* in vivo* [1]. However, less attention has been paid to studies considering the heterogeneity of cellular membrane systems and the highly crowded milieu encountered in cells. For example, it has been shown that hIAPP cytotoxicity highly depends on the location of the peptide. There is an enormous difference if pancreatic *β*-cells are exposed exogenously and endogenously to hIAPP, respectively [61]. Here, we compare the amyloidogenic properties of hIAPP in bulk solution and in the presence of various membrane systems which have been found to drastically modulate fibril formation. An interaction between extracellular islet amyloid fibrils and *β*-cell membrane has already been reported in 1973 [88]. Since then, the membrane disruption hypothesis for cytotoxicity has become the most studied for hIAPP and is thus one major focus of this review, with a special focus on the structural changes that occur in hIAPP upon membrane binding and aggregation as investigated in our laboratory. Moreover, the effects of crowding agents and osmolytes, both important constituents of cellular environments, are discussed.

## 2. Characterization of Monomeric hIAPP and Its Aggregation and Fibrillation Propensity in Bulk Solution

**Figure 3:**
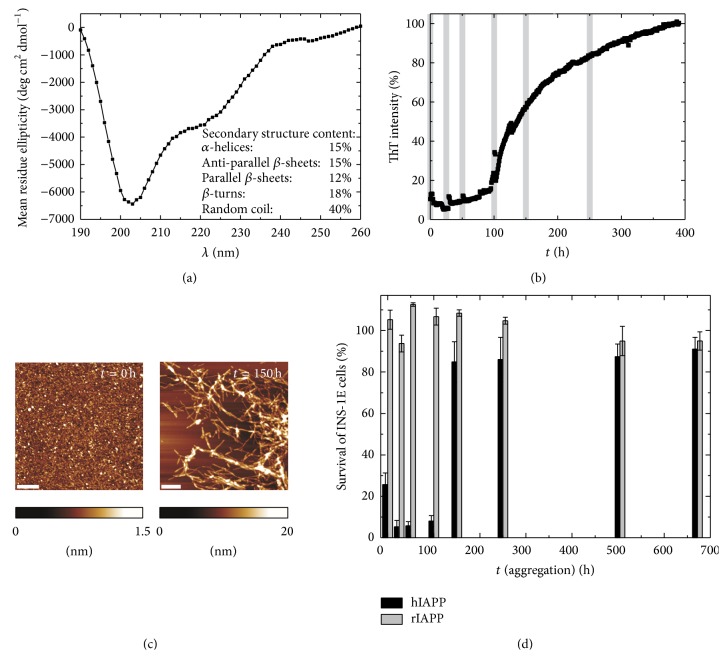
Structure, morphology, and cytotoxicity of hIAPP during its self-assembly. (a) CD spectrum of freshly prepared hIAPP (10 *μ*M) in 10 mM phosphate buffer, pH 7.4 at 25°C, and secondary structure content obtained indicating a predominantly disordered structure of the monomeric peptide. (b) ThT assay of 100 *μ*M hIAPP in 10 mM sodium acetate buffer containing 50 *μ*M ThT, at pH 5.5, and 10°C showing a sigmoidal curve typical for a nucleation-dependent process. The fluorescence intensity was normalized to the intensity recorded at 400 h assuming the fibril formation to be completed. Time points at which hIAPP species were isolated are highlighted in gray. (c) Tapping mode AFM images of isolated hIAPP species at particular time points of the aggregation process showing the hIAPP morphology within the lag phase and the elongation phase. The scale bar included in the images represents 250 nm. (d) WST-1 cell proliferation assay of pancreatic INS-1E cells exposed to 10 *μ*M isolated hIAPP and nonamyloidogenic ratIAPP species at different time points of the aggregation process, indicating the highest toxicity of hIAPP species within the lag phase. Adapted and modified from [17, 56] with permission from Wiley-VCH and Elsevier.

hIAPP is one of the most amyloidogenic peptides. The aggregation kinetics depends on the monomer concentration as well as on the presence of aggregation nuclei and is often too fast to be resolved by spectroscopic methods. One strategy to decelerate the fibrillation time, which is often needed for time-dependent studies, is directly derived from nature. After* in vivo* synthesis, hIAPP is stored in the *β*-cell granules of the pancreas at a pH of approximately 5.5, and, when in need, released into the extracellular compartment at a pH of 7.4. Khemtémourian et al. have shown that low pH decreases the rate of fibril formation [89]. They also showed that these differences in kinetics are directly linked to changes in the conformational behavior of the peptide. One explanation is the protonation of His18, resulting in repulsive interactions between the peptides. In addition, hIAPP is often stored in or pretreated with hexafluoroisopropanol (HFIP) in order to dissolve any form of aggregates and to keep the peptide in its denatured monomeric conformation. Lowering the pH value to 5.5 and pretreatment with HFIP allowed the study of the conformation of monomeric hIAPP by using far-UV CD, FTIR, and NMR spectroscopy. A typical CD spectrum of monomeric hIAPP exhibits a minimum at ~201 nm along with a shoulder around 220 nm, indicating a predominantly disordered structure (~40%) of hIAPP in its initial conformation (Figure 3(a)), which is in good agreement with FTIR data showing an amide-I′ band maximum around 1645 cm^−1^ [56]. The predominant random coil conformation of native hIAPP is also in agreement with literature data [24, 25]. Corresponding results of molecular dynamics (MD) simulations revealed an essentially random-coiled conformation of hIAPP in solution, although transient *α*-helices were observed as well [90]. 2D-NMR spectroscopy data was also employed to elucidate the monomeric structure of hIAPP and the role of specific amino acids. The chemical shift dispersion observed is characteristic for a largely disordered peptide [91]. Solution NMR data [59, 92, 93] also suggest that the monomeric states of hIAPP transiently sample helical states and show a lack of stable secondary structures. Thus, the helical state of hIAPP seems to be a low-lying excited state conformer. Relating to the hIAPP self-association in the bulk phase, time-lapse NMR data strongly suggested that the N-terminal region of hIAPP is involved in the initial step of aggregation, followed by transient *α*-helical intermediate structures [91]. This is consistent with observations that the presence of low percentages of the helix-inducing solvent HFIP strongly catalyzes the aggregation of hIAPP. In another recent MD simulation study on monomeric hIAPP, Singh et al. highlighted the interconversion of hIAPP between an *α*-helix and a *β*-hairpin as an important activating process that could be the initial step of the nucleation process [94].

The extrinsic fluorescent dye thioflavin T (ThT) has been established as a standard tool to follow the fibrillation process of amyloidogenic peptides. ThT displays enhanced fluorescence upon noncovalent binding of mature amyloid fibrils where it binds to *β*-sheet rich areas, probably in a channel binding mode [95, 96]. As an example, Figure 3(b) shows data using the ThT assay for 100 *μ*M hIAPP in acetate buffer, pH 5.5 at 10°C. A lag phase for the first ~100 h followed by a slow exponential growth phase with the fibril formation completed after ~400 h was detected. The morphology of the isolated hIAPP species was analyzed by tapping mode AFM [17]. hIAPP oligomers appeared nearly exclusively between the time points of 0 and 100 h which is the lag phase of the aggregation process. The mean height ± standard deviation of the oligomers detected at 0 h was 0.7 ± 0.2 nm. Exponential growth was observed subsequently in the ThT assay where hIAPP proto-fibrils are formed, as revealed by AFM. These species exhibited a mean height of 3.9 ± 1.0 nm at 150 h of incubation time. After longer aggregation times up to 28 days, higher-ordered *μ*m-long fibrillar structures were detected showing a mean height of 6.4 ± 1.8 nm. Only few oligomeric structures with a mean height of ~0.9 nm remained in the solution.

## 3. Fibrillation Kinetics of hIAPP in the Presence of Lipid Bilayer Membranes

**Figure 4:**
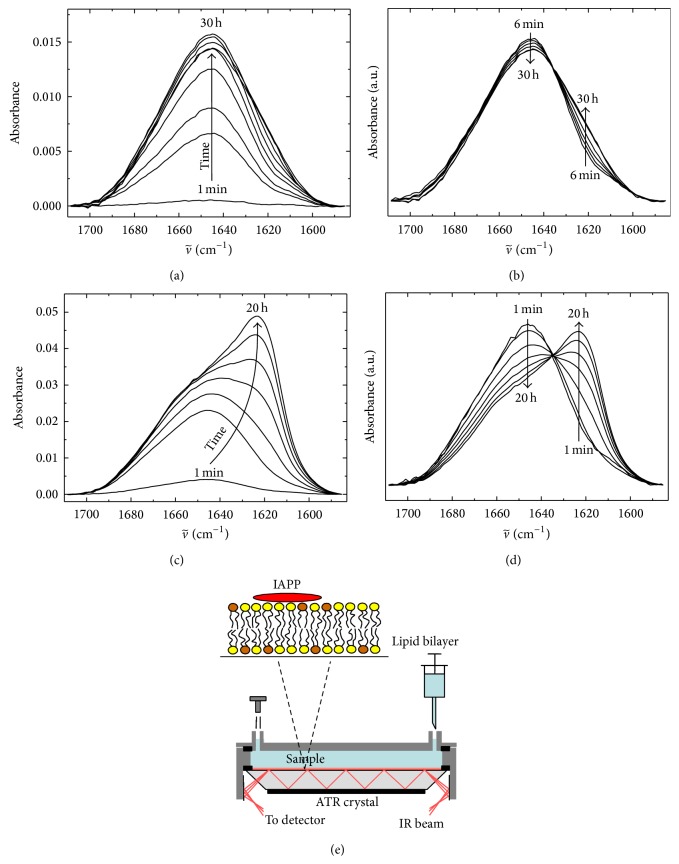
Time evolution of the secondary structural change of hIAPP aggregation by studying the amide-I′ bands at 25°C after injection into the ATR-FTIR cell. (a, b) 10 *μ*M hIAPP in the presence of a neutral (zwitterionic) DOPC bilayer. (c, d) 10 *μ*M hIAPP in the presence of a phospholipid bilayer consisting of DOPC/DOPG (7 : 3, w/w). In (a) and (c), primary ATR-FTIR spectra are shown. In (b) and (d), intensity normalized spectra are depicted. A structural transition from partially *α*-helical and disordered conformations to the formation of intermolecular *β*-sheet occurs only in the presence of anionic lipid bilayers. (e) Schematics of the ATR-FTIR sample cell. hIAPP molecules adsorbing to and aggregating/fibrillating at the lipid bilayer membrane are detected, while those distant from the membrane are merely visible owing to the low penetration depth of the evanescent wave in the ATR-FTIR setup. Adapted and modified from [56] with permission from Elsevier.

The interaction of hIAPP with lipid membranes has been considered to be a main reason for the cytotoxicity of hIAPP. Hence, the properties of hIAPP, while interacting with lipid membranes of different composition, have been extensively studied. In the past, attenuated total reflectance Fourier-transform infrared (ATR-FTIR) spectroscopy has been established in our laboratory as a promising tool to investigate the time-dependent secondary structural changes of hIAPP aggregation in the presence of lipid bilayers. In Figure 4, the aggregation propensity of hIAPP in the presence of a neutral, zwitterionic DOPC and an anionic DOPC/DOPG 7 : 3 w/w lipid bilayers was evaluated by ATR-FTIR spectroscopy. Comparison of the time evolution of the amide-I′ band shows that the presence of DOPC/DOPG strongly favoured the peak shift of the amide-I′ band from 1644 cm^−1^ to 1627 cm^−1^, indicating a decrease in unordered conformations and a concomitant increase in intermolecular *β*-sheet structures. A broad peak at 1616–1619 cm^−1^ appeared during the aggregation process, reflecting the formation of intermolecular *β*-sheets with strong hydrogen bonding [56]. Conversely, no significant aggregation could be observed in the presence of the pure zwitterionic DOPC membrane within 30 h. Only a small shoulder appeared after 20 h in the intermolecular *β*-sheet region around ~1625 cm^−1^, indicating the formation of less ordered, probably oligomeric, aggregate structures after long incubation times.

In many amyloidogenic proteins, small oligomers have been found to form metastable intermediates such as small oligomers which are transiently formed and rapidly converted to amyloid fibrils. By performing sedimentation velocity experiments, Vaiana et al. showed that only <1% of the total population of hIAPP form low-weight oligomers before fibrillation, indicating that once oligomeric aggregation-prone species are formed, they are rapidly utilized in the formation of *β*-rich fibrils [97]. The observation of an essentially all-or-nothing process of hIAPP aggregation can also be found in the ATR-FTIR data (Figure 4(d)), which reveals an isosbestic kind of point between the initial and the final aggregate state, indicating the absence of a large amount of intermediate oligomeric species.

Complementary results have been obtained using infrared reflection absorption spectroscopy (IRRAS) on lipid monolayers. No significant aggregation of hIAPP was observed in the presence of a neutral, zwitterionic POPC lipid monolayer, whereas results for negatively charged POPG monolayers revealed significant fibrillation [57]. Thus, the aggregation process of hIAPP is considerably enhanced in the presence of lipid bilayers with a negatively charged head group. Additional X-ray reflectivity (XRR) and AFM studies confirmed that the accelerative effect is initiated by an electrostatic interaction between the positively charged N-terminal amino acid residues of hIAPP and the negatively charged lipid head groups [98]. Based on the data obtained a multiple step fibrillation mechanism for hIAPP was proposed: First, hIAPP inserts into the membrane through its N-terminus via electrostatic interaction resulting in a conformational transition from a predominantly random coil structure to an *α*-helical conformation. Knight and coworkers showed that membrane binding of IAPP is a cooperative process leading to the formation of membrane-bound and heterogeneous *α*-helical aggregates. The structural conversion of the monomeric IAPP from predominantly disordered to *α*-helical and the subsequent formation of heterogeneous *α*-helical aggregates upon membrane binding hold true for both amyloidogenic hIAPP and nonamyloidogenic ratIAPP [74]. Thereafter, rapid conversion to a *β*-sheet conformation of hIAPP takes place, followed by formation of ordered fibrillar structures. In addition, membrane binding causes a reduced dimensionality and thus an increased local peptide concentration which finally promote the growth of hIAPP fibrils. In a recent MD study by Jia et al. it has been found that the quick adsorption of hIAPP monomers to the lipid bilayer surface is mediated by strong electrostatic interactions of the positively charged residues K1 and R11 with the negatively charged lipid head groups [99]. A stable helix through residues 7–22 realizes a parallel binding of hIAPP to the lipid bilayer surface via electrostatic and H-bonding interactions [100]. This is in agreement with the observation that the fragment hIAPP_20–29_ features a lower affinity to membrane and without any preference for anionic lipids [101]. The facts that hIAPP_1–19_, the nonamyloidogenic fragment, and ratIAPP at high concentrations are able to cause disruption in anionic lipids as well suggest that amyloid formation is not a necessary condition for membrane damage [74, 102]. This is in line with kinetic leakage studies with hIAPP wildtype and mutants by Cao et al. demonstrating that membrane leakage does not require the formation of *β*-sheet or *α*-helical structures [103]. These results would fit to the model of a biphasic kinetics of membrane disruption by hIAPP with distinct fibril-independent and fibril-dependent phases as shown by dye leakage experiments [68, 104]. Interestingly, His18 plays an important role in the orientation of the peptide on the membrane and its protonation, as found in *β*-cell granules, might modulate the membrane disruption effect of hIAPP [103, 105]. However, the exchange of histidine to arginine at position 18 and the lower lipid affinity of ratIAPP at pH 7.4 compared to hIAPP suggest that the electrostatic contribution is not the only factor controlling the membrane binding behavior. A conformational change in the *α*-helix induced by the difference between the membrane-binding domains of hIAPP and ratIAPP might define their membrane affinity [74].

Upon formation, the mature hIAPP fibrils show evidence to detach from the lipid membrane into the bulk solution or they remain adsorbed at the lipid interface. Via direct fluorescence microscopic observation, Domanov and Kinnunen showed that hIAPP fibrillation on the surface of supported lipid bilayers induces deformation, vesiculation and tubulation of the membrane [106]. In addition, hIAPP fibrils have been observed to be coated by lipid membranes derived from the vesicles and tubes. Complementary, Sasahara et al. performed fluorescence recovery after photobleaching (FRAP) measurements and reported on a significant fluidity decrease of model lipid bilayers upon binding of soluble hIAPP, suggesting morphological and functional perturbation caused by the hIAPP-membrane interaction [107].

Plasma membranes are organized in (dynamic) microdomains termed lipid rafts. They play a key role in many biological processes, such as modulating a broad range of signalling cascades [108–110]. Therefore, hIAPP-membrane studies were extended to neutral and anionic heterogeneous membrane systems displaying a coexistence of liquid-ordered (l_o_) and liquid-disordered (l_d_) phase. In our laboratory, a neutral DOPC/DPPC/cholesterol (1 : 2 : 1) and an anionic DOPC/DOPG/DPPC/DPPG/chol (15 : 10 : 40 : 10 : 25) lipid raft mixture, both exhibiting l_o_ and l_d_ phase coexistence were used [111, 112]. Confocal fluorescence microscopy of giant unilamellar vesicles (GUVs) of those lipid mixtures showed that hIAPP rapidly and preferentially partitions into the liquid-disordered (l_d_) domains of the neutral model raft membrane, that is, the domains containing less cholesterol. With time, hIAPP was observed to induce permeabilization of the membrane and disintegration of the GUVs. However, colocalization of hIAPP and the fluid lipid domain was still detectable, indicating an incorporation of lipids into the hIAPP aggregates. After ~72 h of incubation no intact GUVs were detectable anymore [17, 111]. The same systems were investigated by time-lapse tapping mode AFM to yield structural data on a nanometer scale. The results indicated a rapid permeabilizing effect of hIAPP on the zwitterionic lipid raft membrane (DOPC/DPPC/chol, 1 : 2 : 1), accompanied by disruption of the lateral organization of the lipid bilayer within minutes after peptide addition. This degrading effect of hIAPP to the heterogeneous membrane seemed to occur through an unspecific, detergent-like mechanism. Corresponding studies carried out on the anionic lipid raft membrane (DOPC/DOPG/DPPC/DPPG/chol, 15 : 10 : 40 : 10 : 25) showed an even more accelerated kinetics than for the aggregation in the presence of the homogeneous anionic lipid membrane. Complementary ATR-FTIR studies revealed a slower aggregation kinetics of hIAPP in the presence of the neutral heterogeneous membrane compared to the scenario with 30% anionic membrane.

Taken together, these data clearly demonstrate that the hIAPP-membrane interaction is more pronounced at anionic membranes since a stronger adsorption of hIAPP to both the homo- and heterogeneous anionic membrane compared to the neutral membrane systems was observed. The electrostatic interaction between the positively charged N-terminal amino acid residues of hIAPP and the negatively charged lipid head groups were found to be a dominating effect causing the peptide-membrane interaction. However, whereas hIAPP does not seem to aggregate substantially in the presence of the homogeneous zwitterionic membranes, significant aggregation at heterogeneous zwitterionic bilayers takes place as well, as also found for other raft containing membrane systems [113]. The rapid initial adsorption of hIAPP at defect states, such as the rim of the coexisting l_o_ and l_d_ lipid domains, may be the reason for an increased local peptide concentration at the heterogeneous membrane which leads to an enhanced fibrillation even in the absence of charged head groups. Next to the lipid bilayer's lateral organization, also the constituting lipid components influence the aggregation propensity of hIAPP. For example, phosphatidylethanolamine (PE), a lipid with an intrinsic negative curvature, has been shown to hamper the fibril-independent phase of membrane disruption, but enhances the membrane leakage correlated with the growth of fibrils on the membrane surface via a detergent-like mechanism [114]. Cholesterol has been found to effectively modulate hIAPP fibrillation as well [115, 116].

## 4. Interaction of hIAPP with Biological *β*-Cell Model Membranes

**Figure 5:**
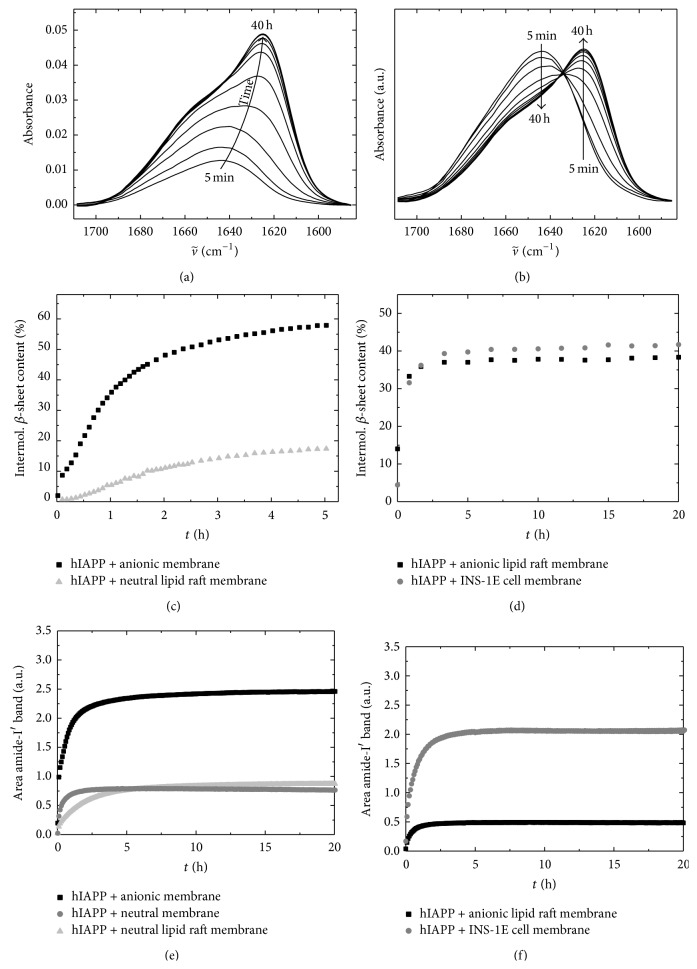
Aggregation kinetics of hIAPP in the presence of different membrane models. Time evolution of the amide-I′ bands of 3 *μ*M hIAPP upon aggregation in the presence of a membrane composed of lipids extracted from a pancreatic *β*-cell line of rat (INS-1E) at 25°C. In (a), primary ATR-FTIR spectra are shown; (b) depicts the concomitant intensity normalized spectra. (c–f) Time evolution of the *β*-sheet content and adsorption kinetics upon aggregation of hIAPP in the presence of various membrane compositions. (c, e) 10 *μ*M hIAPP at an anionic (DOPC/DOPG, 7 : 3, w/w) membrane, a neutral, zwitterionic DOPC membrane and a neutral heterogeneous lipid raft membrane (DOPC/DPPC/chol, 1 : 2 : 1). (d, f) 3 *μ*M hIAPP in the presence of an anionic heterogeneous lipid raft membrane (DOPC/DOPG/DPPC/DPPG/chol, 15 : 10 : 40 : 10 : 25) and a membrane composed of lipids extracted from the pancreatic *β*-cell line of rat INS-1E. Adapted and modified from [56, 60] with permission from Elsevier.

Model lipid bilayers consisting of few components are often insufficient in order to represent the scenario* in vivo*. Therefore, our laboratory extracted cell membrane lipids from a pancreatic *β*-cell line of rat (INS-1E) [117] to be able to study the hIAPP-membrane interaction in a more natural lipid environment. Mass spectrometry analysis of the extracted lipids revealed phosphatidylcholine (PC) as the major head group component of the lipid mixture and a ratio of 2.5% negatively charged lipids [60]. The ATR-FTIR spectroscopy data show that hIAPP adsorbs readily at the membrane and shows an increasing amide-I′ band intensity at around 1623 cm^−1^—indicating intermolecular *β*-sheet formation—already 1 h after the measurement was started (Figure 5). These findings are similar to those obtained for hIAPP aggregation and fibrillation at the homo- and heterogeneous anionic lipid membranes. However, a much stronger adsorption of hIAPP to the biological model membrane was detected (Figures 5(b) and 5(f)). This could be explained by a higher roughness of the biological membrane with a higher concentration of membrane defects which could foster the interaction of hIAPP with the membrane surface.

**Figure 6:**
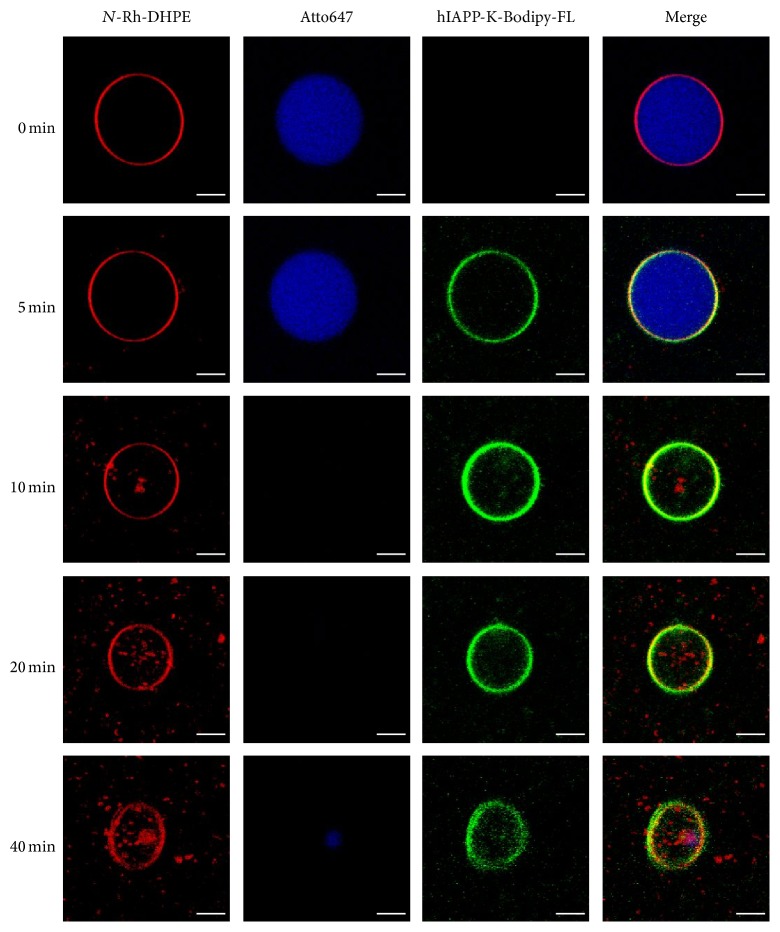
Visual leakage assay studying the interaction of hIAPP with biological membrane and its consequence. Confocal fluorescence microscopy images of the interaction of 5 *μ*M hIAPP with giant unilamellar vesicles (GUVs) composed of lipids extracted from a pancreatic *β*-cell line of rat (INS-1E). The GUVs which are labelled with* N*-Rh-DHPE (red) are filled with phosphate buffer containing the fluorophore Atto647 (blue). C-terminally labelled hIAPP-K-Bodipy-FL (green) adsorbs within the first 5 min to the lipid vesicles and leads to membrane permeabilization and disintegration of the GUV. The scale bars represent 10 *μ*m. Reprinted from [60] with permission from Elsevier.

Fluorescence microscopy measurements on giant unilamellar vesicles (GUVs) of the extracted biological lipids from the *β*-cell membrane supported these findings. To detect the permeabilization of the vesicles upon hIAPP-induced disintegration of the lipid membrane, a leakage test was employed. The GUVs were filled with buffer containing the fluorophore Atto647. The lipids were labelled by addition of* N*-Rh-DHPE (*N*-(lissamine rhodamine B sulfonyl)-1,2-dihexadecanoyl-*sn*-glycero-3-phospho-ethanolamine triethylammonium salt). For visualization of hIAPP, the peptide was C-terminally labelled with Bodipy-FL and a 5 *μ*M solution of hIAPP-K-Bodipy-FL was added to the GUVs. Fluorescence microscopy images of the interaction are depicted in Figure 6. At first (*t* = 0 min) the GUVs are shown before hIAPP was added, to visualize the Atto647 fluorophore containing buffer (blue channel) within the* N*-Rh-DHPE labelled GUVs (red channel). Already after 5 min, hIAPP-K-Bodipy-FL (green channel) could be mainly detected at the lipid membrane of the vesicles and led within the next minutes to permeabilization and leakage of the membrane. However, colocalization of hIAPP and the biological membrane was still detectable even after *t* = 40 min. At this time point, disintegration of the GUVs was observed, indicating incorporation of lipids into the growing hIAPP aggregates [60].

Taken together, the rapid permeabilization and disintegration of GUVs observed and induced by soluble hIAPP confirm a fibril-independent mechanism of membrane disruption. Subsequent GUV disintegration and lipid incorporation into the hIAPP aggregates give evidence for a second fibril-growth dependent mechanism of membrane disruption, which is in agreement with literature data [68, 75]. One reason for the fibril-independent mechanism could be the hIAPP-insertion induced formation of negative curvature within lipid bilayers as Smith et al. concluded from studies using bicelles [118]. hIAPP- and PE-induced curvature effects may be expected to feature different geometries and energetics, however. The intrinsic negative curvature of PE containing membranes hampers a deep insertion of the peptide into the membrane and favors a shallow binding of amyloid fibers onto the membrane. Hence, insertion of the peptide into the membrane and thus the fibril-independent induced membrane disruption seem to sensitively depend on the geometry and curvature elastic stress of the membrane [114]. Upon fibril growth, further curvature might be induced by the twist of the *β*-sheets of the hIAPP fibrils [69]. In addition, lipid extraction from the lipid bilayers has been observed during fibril growth which might be a second reason for the fibril-dependent membrane damage [119]. The “pore model” for the fibril-independent membrane leakage has been put forward by Last and Miranker [120]. Using the amphipathic peptides magainin 2 and ratIAPP, they pointed out that initial binding of the peptide in the intermediate region between the head group and acyl chains of the bilayer, expands the head group region relative to the acyl region of the membrane. This results in a thinning of the acyl chain region and thus the formation of an internal surface tension within the bilayer due to the nonideal packing of the acyl chains. Formation of membrane pores could be an energetic consequence to release the surface tension. Recent imaging total internal reflection-fluorescence correlation spectroscopy (ITIR-FCS) studies suggest a “carpet model” and showed that below the critical concentration for peptide aggregation and upon binding to the plasma membrane of living cells, monomeric hIAPP increases the fluidity of the plasma membrane by carpeting the plasma membrane and forming microdomains. Such dynamic microdomains, presumably consisting of peptide-lipid complexes, are able to extract lipids in a hIAPP-concentration dependent manner [121, 122].

## 5. Cytotoxicity of hIAPP Polymorphs to Pancreatic *β*-Cells

*In vivo*, stability and function of proteins are tightly controlled by a protein control network, including chaperone-mediated folding and degradation of misfolded proteins via proteasome and autophagy. Its deficiency results in accumulation of misfolded proteins and causes protein misfolding diseases including T2DM. The mechanism of hIAPP-induced cytotoxicity is not yet fully understood, but it seems to cause dysfunctions at different cellular and subcellular levels including production of ROS, ER-stress, defects in UPS and autophagy, increased production of proinflammatory cytokines, and in particular permeabilization of plasma and mitochondria membranes [1]. The hypothesis of intermediate species such as oligomers gaining toxic functions and causing degeneration of functional cells is common [9–12]. However, such oligomers are metastable, probably polydisperse in nature, and thus ill-defined.

Using the pancreatic *β*-cell line INS-1E and a WST-1 cell proliferation assay, our laboratory studied the cytotoxicity of various hIAPP species. The results of the proliferation assay clearly show a correlation between the hIAPP cytotoxicity and the aggregation time [17]. Within the lag phase of hIAPP aggregation, the cells showed only small survival rates between 3.5 and 10% where predominantly oligomeric hIAPP species have been found by AFM experiments. Proceeding of the growth reaction led to an increase in ThT intensity correlated with a significant decrease in cytotoxicity. Samples taken within this exponential growth phase contained mainly proto-fibrils as detected by AFM and showed an 80–85% survival rate of INS-1E cells. Mature hIAPP fibrils, as found in the saturation phase of hIAPP aggregation and confirmed by AFM, exhibited with 90% cell viability the lowest comparative cytotoxicity. As a control, samples at different time points after incubation with ratIAPP were taken. At same concentration, the nonamyloidogenic ratIAPP did not show cytotoxicity within the whole incubation period (Figure 3(d)). However, using the MTT assay (loss of mitochondrial activity) and elevated peptide concentrations or another cell line, ratIAPP has been reported to be also cytotoxic [61, 123]. These results are in agreement with the biphasic mechanism of IAPP-induced membrane disruption found* in vitro*. Soluble hIAPP and even ratIAPP, either monomeric or oligomeric, binds to the membrane surface by electrostatic and hydrophobic interactions. Once initial binding is achieved, further peptides are recruited and growth of hIAPP fibrils is promoted. Both IAPP binding (fibril-independent) and fibril growth of hIAPP lead to membrane disruption and finally cell death. Mature fibrils were shown to be least cytotoxic, which is consistent with* in vitro* data showing that preformed fibrils do not cause membrane disruption [68]. However, the hIAPP cytotoxicity cannot be simply scaled-down to a membrane-disruption phenomenon. Using nontoxic and nonamyloidogenic IAPP mutants, Cao et al. demonstrated that there is no one-to-one relationship between disruption of model membranes and induction of cellular toxicity [103]. Additional contributions might be involved.

Obviously, the typical extracellular amyloid deposits found in T2DM play a minor role in cytotoxicity. In contrast, small and structurally ill-defined oligomers might play a more prominent role in the development of T2DM. For example, recent data illustrate that hIAPP cytotoxicity is correlated with mitochondrial dysfunction upon abnormal intracellular release of toxic hIAPP oligomers from *β*-granules [61, 66]. As reason, a disruption of the mitochondrial membrane integrity is proposed.

## 6. Characterization and Inhibition of hIAPP Fibrillation under Macromolecular Crowding Conditions

Protein aggregation and fibrillation occur naturally in a densely crowded, viscous, and heterogeneous solvent, namely, the cytoplasm, which is filled up to a volume of 40% by differently sized biomolecules such as proteins, nucleic acids, osmolytes, and salts [124]. The consequential reduced accessible volume, also known as the macromolecular crowding effect, is predicted to have a significant impact on the equilibria and kinetics of biochemical processes by limiting the conformational sampling space to maximize the overall available volume [125]. In other words, macromolecules restrict the available molecular space by their mutual impenetrability and repulsive interactions (termed as excluded volume effect) and thus entropically stabilize proteins by favoring the more compact conformations of the protein. The excluded volume effect strongly depends on the relative sizes and shapes of the test molecule and the background macromolecules. The more comparable the crowded molecule and the cosolutes are, the more the excluded volume effect dominates. However, the excluded volume effect is accompanied by an increase of viscosity, reduced dynamics, and changes in solvent polarity and water activity, which in total describes the macromolecular crowding effect. Since in-cell techniques are rare and limited, inert and water soluble synthetic polymer crowding agents such as Ficoll, PEG or dextran have been used to mimic macromolecular crowding conditions and to study the effect of steric repulsion on equilibria of different cellular processes, such as protein folding and aggregation. Ficoll is a copolymer of sucrose and epichlorohydrin and behaves as compact and rigid spheres due to its high branching and cross-linking. In contrast, dextran is a polymer of D-glucopyranose with less branching and thus features high flexibility and linearity typical for a quasi-random coil. Due to their hydrophilicity, inertness, and neutrality, both polymers have been established as well-suited macromolecular crowding agents to mimic biological fluids, whether intra- or extracellular [126]. Despite its high water-solubility, polyethylene glycol (PEG), a linear polymer of ethylene glycol, is a less promising macromolecular cosolute, since evidence of attractive interactions between PEG and hydrophobic side chains on the protein surface has been found [127–129]. More recently, the macromolecular crowding effect includes also the consideration of nonspecific (“soft”) interactions between solute and cosolutes, which are of enthalpic nature and can have stabilizing and destabilizing effects [130–135]. Therefore, in order to mimic the in-cell scenario with more biologically relevant cosolutes, proteins such as BSA and lysozyme were introduced as proteinaceous crowding agents because they feature chemically heterogeneous surfaces, providing chemical interactions such as hydrophobic and electrostatic interactions as well as hydrogen bonding. BSA has a molecular mass of 66.4 kDa and is negatively charged at physiological conditions, whereas the 14.3 kDa lysozyme has a positive net charge.

Many studies reported on the stabilizing effect of* in vitro* macromolecular crowding on protein folding which ranges from modest to strong [125], whereas cellular crowding was shown to only weakly shift protein folding equilibria towards the folded state [136–138] or even to destabilize the native state, such as of the surface antigen VlsE [139].

Over the last decades, effects of macromolecular crowding have slowly gained attention in protein aggregation and fibrillation studies [140]. It was shown that the aggregation and fibrillation reaction of several IDPs, including *α*-synuclein [141–145], A*β* [146], apolipoprotein C-II [147], prion protein (PrP) [148–150], and copper-zinc superoxide dismutase (SOD1) [150], are accelerated by synthetic and protein crowders. In order to understand the accelerative effect of macromolecular crowding on the protein fibrillation reaction, several major steps within the aggregation pathway which might be affected, have to be considered: (1) the structural collapse of the monomer producing aggregation-prone partially folded species, (2) formation of dimers and oligomers inducing the nucleation process, and (3) the growth and elongation of fibrils. The excluded volume effect is expected to favor the first two steps due to structural compaction upon formation, whereas the viscosity of such crowded solutions might decelerate the diffusion-limited process of fibril elongation. By studying the morphology of fibrils from the IDPs described above and formed under macromolecular crowding conditions, increased amounts of fibrils featuring shorter lengths have been reported. This indicates that the excluded volume effect favors the steps of structural transformation within the monomer and the oligomer formation, which dominate over the viscosity effect of macromolecular crowding. However, the net effect of macromolecular crowding on protein fibrillation might be different for different IDPs.

**Figure 7:**
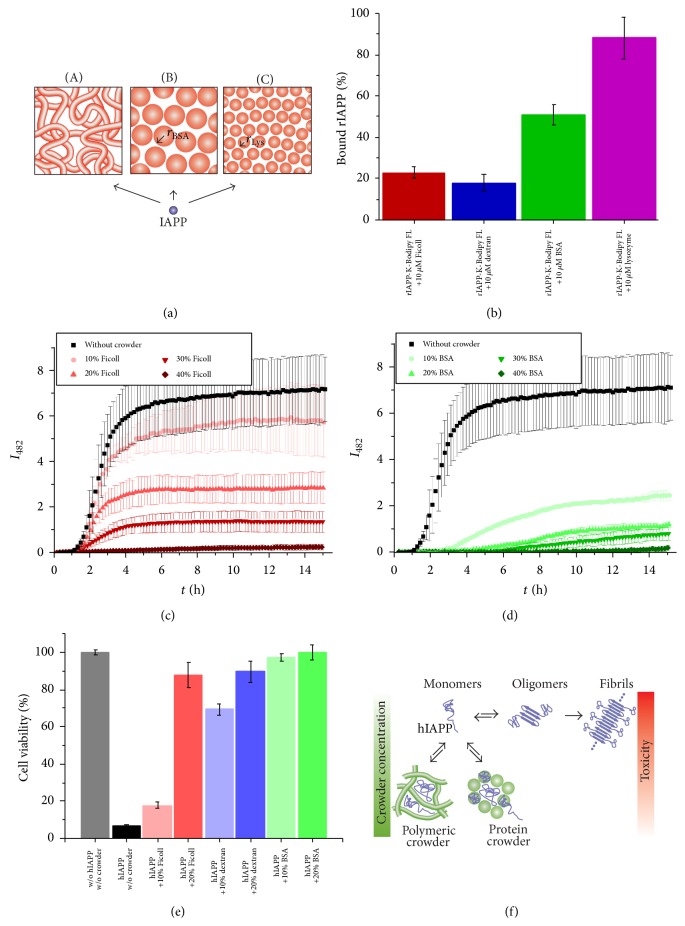
Suppressive effect of macromolecular crowding on hIAPP aggregation and cytotoxicity. (a) Schematic illustration of the excluded volume effect caused by different polymer and protein crowding agents (A: Ficoll and dextran, B: BSA, C: lysozyme). (b) Moderate to strong interaction between ratIAPP and the crowding agent at equimolar concentration, as analyzed from FCS measurements, affect the aggregation kinetics of 10 *μ*M hIAPP differently as measured by ThT fluorescence spectroscopy (c, d). In addition, a crowder concentration dependent decrease of the fibril amount formed is observed. (e) The effect of macromolecular crowding favors the formation of nontoxic off-pathway hIAPP species. (f) In summary, with the results from complementary AFM and ATR-FTIR studies, two competing reaction pathways of hIAPP aggregation under crowding conditions are revealed. The horizontal path shows the well-known hIAPP aggregation mechanism from a natural monomeric disordered structure via formation of nuclei and oligomers to fibril accumulation. The competing crowder type dependent stabilization of nontoxic, off-pathway and globular hIAPP species is shown in a vertical path. Adapted and modified from [151].

Since hIAPP fibrillation occurs naturally in crowded biological fluids, both intra- and extracellular, our laboratory recently analyzed the influence of macromolecular crowding on the aggregation properties of hIAPP by using different polymeric (dextran 70, Ficoll 70) and protein (BSA, lysozyme) crowding agents (Figure 7). In contrast to previous studies on IDPs, we found suppressive effects of macromolecular crowding on the hIAPP fibril formation [151, 152]. First, by applying fluorescence correlation spectroscopy (FCS) we found that the mobility of monomeric IAPP was highly restricted by interaction with the cosolute and increased viscosity under such macromolecular crowding conditions. 20% of ratIAPP was found to bind to the polymeric crowding agents Ficoll and dextran, whereas in solution with BSA nearly 50% of ratIAPP and with lysozyme nearly 90% of ratIAPP were bound to the protein crowder (Figure 7(b)). By applying ThT fluorescence spectroscopy (Figures 7(c) and 7(d)) and AFM, a crowder-type and concentration dependent decrease of fibril formation was observed. However, for all concentrations of Ficoll and dextran the aggregation kinetics of hIAPP remained nearly unchanged, except for higher amount of dextran (30–40 wt%), resulting in a slightly extended elongation phase. In contrast, the protein crowders caused a prolonged lag phase and an extended elongation phase in case of BSA and a complete inhibition in the presence of 10 wt% lysozyme. Additional data gained by ATR-FTIR spectroscopy showed that the structural transition from partially *α*-helical and disordered in the monomeric state to the formation of intermolecular *β*-sheets in the aggregated state remained unchanged under macromolecular crowding conditions. The well-known accelerative effect of lipid environment on hIAPP fibrillation was not affected by macromolecular crowding. Taken together, these observations confirmed that the* in vitro* hIAPP aggregation pathway from monomeric species to fibril formation via nucleation is competing with stabilization of early-stage hIAPP species under macromolecular crowding conditions by nonspecific binding between structurally disordered IAPP monomers and crowding agents and increased viscosity. This study showed that the cellular crowding might not only be an effect of excluded volume, at least for hIAPP, but also strongly involves nonspecific interactions (enthalpic contribution). Compared to the other IDPs studied under macromolecular crowding conditions, hIAPP is the smallest peptide with only 37 amino acid residues and thus is rather insensitive to the excluded volume effect. The cavities confined by the cosolutes might be still too large in order to favor the structural transition and nucleation of hIAPP as expected from the excluded volume effect. In a second study, we compared the modulation effect of the macromolecular crowding agent Ficoll 70 and its monomeric equivalent sucrose on the aggregation reaction of hIAPP. We found that both have approximately the same suppressive effect on the aggregation kinetics and the amount of fibrils formed confirming that Ficoll behaves osmolyte-like and that the steric excluded volume effect does not have a major effect on hIAPP aggregation. Lee et al. reported on inhibitory effects of Ficoll and promoting effects of dextran as crowding agents on the fibrillation reaction of A*β*, a comparable peptide in size and involved in Alzheimer's disease, under nonagitating conditions [146]. These results revealed that the chemical nature and the increased viscosity of a crowding solution can determine the macromolecular crowding effect and thus dominate over the steric effect of excluded volume. Computer simulation studies of our laboratory showed complementary results for fragments of hIAPP. The probability of the aggregated state vanishes upon decreasing the system size suggesting that the finite size of biological cells or their compartments may be playing a key role in hampering intracellular aggregation of highly amyloidogenic peptides, whereas aggregation occurs more frequently in lower crowded environments, such as the extracellular space [153].

Recently, Huang et al. reported on the stabilization of soluble and neurotoxic *β*-oligomers of the recombinant human prion protein (PrP^C^) under macromolecular crowding conditions [148]. In contrast, we found that the globular, early-stage hIAPP species stabilized under macromolecular crowding conditions were not toxic when exposed exogenously to the pancreatic *β*-cell line INS-1E, indicating that those species are formed off-pathway (Figure 7(e)).

Low-concentration working small molecules become attractive in the light of therapeutic applications. Mostly, they inhibit protein aggregation and subsequent fibrillation by direct and hydrophobic interaction due to their planar structure and hydrophobic nature. Thereby, they bind to partially folded regions within the monomer, lead to formation of nontoxic off-pathway oligomers, or destabilize the fibrillar states upon binding to mature fibrils [154]. However, for the development of such pharmacological chaperones, it is also crucial to consider the effect of macromolecular crowding on its inhibition efficiency since the biological fluid itself has a significant regulative effect on protein aggregation and fibrillation* in vivo*. Therefore, we characterized and compared the effect of an orcein-related small molecule, O4, which has been shown to reduce the cytotoxicity of A*β* by stimulating its aggregation [155], on the hIAPP fibrillation in the absence and presence of crowding agents [152].* In vitro*, we have shown that O4 is an efficient dose-dependent inhibitor of hIAPP aggregation by redirecting the aggregation pathway towards off-pathway globular species, thereby reducing the cytotoxicity of hIAPP. Further, O4 is able to interact with hIAPP fibrils at a mature stage, causing disassembly of fibrils into smaller, less stable structures. A less effective inhibitory effect of O4 was found in the presence of Ficoll 70, whereas in the presence of its monomeric unit sucrose, the dose-dependent inhibitory effect of O4 is similar to that in the diluted buffer, indicating that macromolecular crowding does modulate the efficiency of O4.

Taken together, those studies clearly demonstrate that the biological fluid itself might be an active contributor regulating the amyloidogenic propensity of hIAPP* in vivo*. An increase of water content and thus a decrease of macromolecular crowding, as found in the extracellular space or induced by a loss of the health status of the cell, might be a reason for the onset of hIAPP aggregation* in vivo*. In addition, the data highlights the importance and need to develop in-cell methods in order to get insight into the mechanism of hIAPP fibrillation in living cells where the influence of biomolecular solvation, viscosity, excluded volume and complex membrane systems act in concert. The recently developed optical super-resolution techniques such as stimulated emission depletion (STED) and stochastic optical reconstruction microscopy (STORM) might be promising tools to spatiotemporally resolve structural and morphological properties of hIAPP inside living cells. Schierle et al. used direct STORM to successfully show the fibrillar structure of* in situ* formed A*β* aggregates [156].

## 7. Modulation of hIAPP Aggregation and Fibrillation by Osmolytes

Apart from macromolecules such as proteins, nucleic acids and lipids, the cellular cytoplasm contains additional small organic molecules, also known as osmolytes or chemical chaperones. They function as responders to equilibrate cellular osmotic pressure and thus to maintain protein stability and functionality [157–159]. Depending on how they affect the folding equilibrium of a protein, the osmolytes can be divided into two classes, namely denaturants and protecting (or compatible) osmolytes. Denaturants such as urea and guanidinium chloride favor the unfolded states of proteins by forming intermolecular hydrogen bonds with the peptide backbone and polar side chains, which are more favored than intramolecular hydrogen bonds required for the formation of secondary and tertiary structures [157]. In contrast, protecting osmolytes including carbohydrates (e.g., trehalose), polyoles (e.g., glycerine, sorbitol, inositol), amino acids (e.g., glycine, proline, and taurine), and methylamines (e.g., TMAO, glycine-betaine) stabilize protein's native structure by favoring interaction with the solvent water and excluding themselves from the protein surface [157]. As a consequence, this preferential exclusion effect (also termed as osmophobic effect) shifts the folding equilibrium towards more compact structures with less solvent accessible surface area (SASA) [160–162]. In nature, denaturants and protecting osmolytes such as urea and TMAO often act in a certain ratio in concert to be able to regulate the protein stability under varying osmotic and other environmental conditions [157].

It is still poorly understood how osmolytes affect the stability of aggregation-prone proteins and thus their aggregation propensity. In literature, the rarely investigated osmolyte effect ranges from aggregation-inhibiting to aggregation-inducing. At a closer inspection, those conflicting results derive from the protein's specific tendency to aggregate. Chemically or mutation-induced protein destabilization leads to partial unfolding and thus formation of aggregation-competent species that can be ameliorated in the presence of osmolytes such as sorbitol, glycine-betaine and trehalose [163–165]. In contrast, fibrillation of natively unfolded proteins such as IDPs has been reported to be enhanced and accelerated in the presence of osmolytes such as TMAO, glycerine or glycine-betaine [166–169]. The latter observation indicates stabilization of compact structures, probably aggregation-competent nuclei, whose free energy is decreased due to the osmophobic effect.

**Figure 8:**
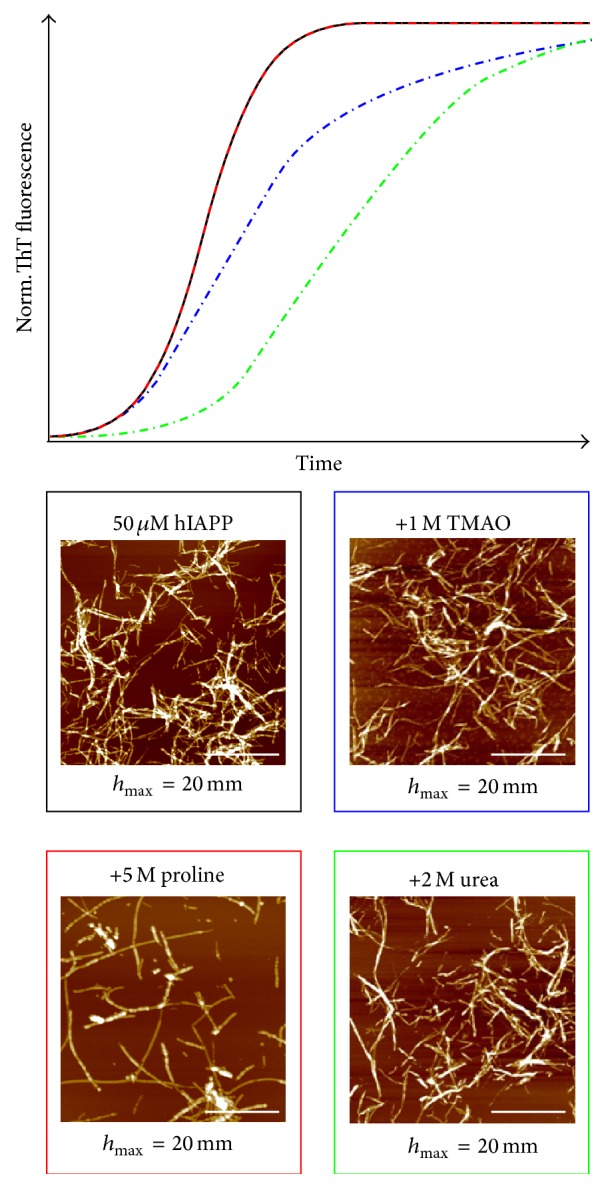
Effect of various osmolytes on the aggregation kinetics and morphology of hIAPP. Schematic summary of ThT fluorescence assays shows the effect of TMAO (blue), proline (red) and urea (green) on the aggregation kinetics of hIAPP. The corresponding effect on the morphology of the mature fibrils is studied by AFM. All scale bars indicate 1 *μ*m. Adapted and modified from [152, 170] with permission from the Royal Society of Chemistry.

Recently, our laboratory investigated the effect of TMAO, glycine-betaine, proline, and urea on the fibrillation reaction of hIAPP [152, 170]. ThT fluorescence and ATR-FTIR spectroscopy data revealed a TMAO- and glycine-betaine-induced stabilization of hIAPP proto-fibrils causing retardation of fibril elongation, whereas AFM images showed that the morphology of the mature fibrils were not affected by those osmolytes. Despite the similar outcome, the mode of action of TMAO and glycine-betaine differed from each other. The prolongation effect of TMAO was concentration-dependent, whereas the same effect of glycine-betaine was concentration-independent. In case of the denaturant urea, a concentration-dependent prolongation of the lag phase has been observed, indicating stabilization of hIAPP in an aggregation-incompetent state and retardation of IAPP nuclei formation. Interestingly, that effect was fully compensated by adding TMAO in a molar ratio of 2 : 1 urea : TMAO, which could not be found for glycine-betaine indicating direct interaction between TMAO and urea via hydrogen bonding. For the natural amino acid proline, we found a weak concentration-dependent retardation of the elongation phase as well as a dose-dependent decrease of the amount of hIAPP fibrils formed. AFM measurements revealed shortening of hIAPP fibrils and formation of globular, amorphous aggregates apart from the fibrillar assemblies in the presence of proline (Figure 8). This suggests that proline diverts the amyloidogenesis of hIAPP into an alternative aggregation pathway where shorter and smaller, fibrillar and nonfibrillar species are formed. The observation of disfavoring fibril formation is consistent with the hypothesis of proline being excluded from the protein's surface. As a response to the preferential exclusion effect, hIAPP might form nonfibrillar aggregates in order to minimize the exposed surface area.

Studies of Auton and Bolen determined TMAO as the most effective protecting osmolyte followed by glycine-betaine and proline when the transfer free energy values of peptide backbone units from 1 M osmolyte to water were considered [171, 172]. Such a trend could be observed for the modulation of hIAPP fibrillation. TMAO and glycine-betaine stabilize hIAPP in its proto-fibrillar state, whereas proline shifts the equilibrium of hIAPP aggregation towards formation of amorphous assemblies. However, the results also imply that additional interactions between the peptide and the osmolyte are involved apart from the preferential exclusion effect in order to subtly modulate the osmolyte effect of hIAPP aggregation. Evidence can be found also in the literature by comparing the TMAO effect on different IDPs. The fibrillation reaction of tau, *α*-synuclein, and A*β* has been reported to be accelerated in the presence of TMAO, whereas TMAO induces the formation of amorphous aggregates in case of the glutamine-rich IDP huntingtin exon 1 [173]. Interestingly, despite a 25% identity and 50% similarity between A*β* and hIAPP in their primary structure and a cross-aggregation ability with hIAPP [60, 174], TMAO acts differently on their aggregation reaction. Taken together, the results clearly show that osmolytes do not only modulate the stability, but also the aggregation propensity of IDPs inside cells. However, the modulation outcome highly depends on the structural and chemical properties of the monomeric species.

## 8. Conclusions

To conclude, we have shown that by studying specific steps in the aggregation and fibrillation process of hIAPP, such reductionist biophysical approach can yield useful information on the very complex behavior of hIAPP at the molecular level, which might also contribute valuable insights into the mechanisms by which the amyloidogenic peptide may induce cell toxicity. However, as no simple relationship between the disruption and IAPP-induced leakage of membranes and cellular toxicity has been found, additional factors may seem to play a role as well. Moreover, there must be factors operating* in vivo* that attenuate the otherwise strong amyloidogenic propensity and membrane disruptive characteristics of hIAPP found* in vitro*. Acidic conditions [79], divalent ions (Ca^2+^ [175], Zn^2+^ [85, 104]) and interaction with insulin [18, 113, 176] have been reported to strongly reduce hIAPP's amyloidogenesis and membrane disrupting propensity. In addition, we demonstrated in this review that ubiquitous effects in cells such as macromolecular crowding and the presence of osmolytes have significant regulative and even suppressive effects on hIAPP aggregation. However, in future studies more complex and physiologically relevant models including in-cell studies are needed in order to uncover all mechanistic aspects of hIAPP's cellular toxicity. Particularly, a molecular understanding how obesity and aging correlate with the onset of hIAPP aggregation* in vivo* has to gain more attention. Such studies are essential for the development of therapeutic strategies to prevent the age-related and metabolic disease T2DM.

## Abbreviations

TMAO: Trimethylamine N-oxide
DOPC: 1,2-Dioleoyl-*sn*-glycero-3-phosphocholine
DOPG: 1,2-Dioleoyl-*sn*-glycero-3-phospho-*rac*-(1′-glycerol)
DPPC: Dipalmitoylphosphatidylcholine
DPPG: 1,2-Dipalmitoyl-*sn*-glycero-3-phosphoglycerol
POPC: 1-Palmitoyl-2-oleoyl-*sn*-glycero-3-phosphocholine
POPG: 1-Palmitoyl-2-oleoyl-*sn*-glycero-3-phospho-(1′-*rac*-glycerol)
chol: Cholesterol.
